# Taxonomic studies of *Glochidion* (Phyllanthaceae) from the Indo-China Peninsula (I): *G. shanense*, a new species from Myanmar

**DOI:** 10.3897/phytokeys.96.24497

**Published:** 2018-03-27

**Authors:** Gang Yao, Jie Cai, Youheng Wu, Xuefei Yang, Thaung Naing Oo, Aung Zaw Moe, Shixiao Luo

**Affiliations:** 1 South China Limestone Plants Research Centre, College of Forestry and Landscape Architecture, South China Agricultural University, Guangzhou 510642, China; 2 Plant Germplasm and Genomics Centre, Germplasm Bank of Wild Species, Kunming Institute of Botany, Chinese Academy of Sciences, Kunming, 650201, China; 3 Key Laboratory of Plant Resources Conservation and Sustainable Utilisation, South China Botanical Garden, Chinese Academy of Sciences, Guangzhou 516650, China; 4 University of Chinese Academy of Sciences, Beijing 1000049, China; 5 Key Laboratory of Economic Plants and Biotechnology and Yunnan Key Laboratory for Wild Plant Resources, Kunming Institute of Botany, Chinese Academy of Sciences, Kunming 650201, China; 6 Southeast Asia Biodiversity Research Institute, Chinese Academy of Sciences, Yezin, Nay Pyi Taw 05282, Myanmar; 7 Forest Research Institute, Forest Department, Ministry of Environmental Conservation and Forestry, Yezin, Nay Pyi Taw 05282, Myanmar

**Keywords:** Phyllanthaceae, *Glochidion*, Morphology, Myanmar

## Abstract

Based on morphological studies performed on live plants in the field and specimens deposited in herbaria, a new species, *Glochidion shanense* Gang Yao & Shixiao Luo (Phyllantheae, Phyllanthaceae), is here described and illustrated. The species is morphologically most similar to *G. ellipticum* Wight, but can be distinguished from the latter by having hairy branchlets, longer pedicels, uniseriate and narrowly triangular sepals of female flowers, 4–5-locular ovaries, stout and cylindric persistent style on fruits.

## Introduction

*Glochidion* J.R. et G. Forst. is the second largest genus in the tribe Phyllantheae Dumortier, Phyllanthaceae Martynov (Webster 1994). It consists of more than 300 species mainly distributed in the Indo-Pacific, east to southeast Polynesia and south into Australia (Govaerts et al. 2000, Li and Gilbert 2008) and can be distinguished from other members in Phyllantheae by the absence of a floral disc, apiculate anthers, usually confluent styles and fleshy seed-coat (Li 1994, Webster 1994).

Recently, the leafflower plant and leafflower moth system, a new mutualism model system that is similar to the fig-fig wasp and yucca-yucca moth pollination systems, was described between Phyllantheae plants and *Epicephala* moth (Kato et al. 2003, Kawakita et al 2015, Luo et al. 2017) and it provides a new opportunity to study the mechanisms of biodiversity development and maintenance. *Glochidion* is the largest host plant lineage within this newly described mutualism system, in which at least five host plant lineages were identified (Kawakita and Kato 2009). However, a comprehensive taxonomic study of *Glochidion* is still lacking, especially for relevant species distributed in the Indo-China Peninsula, since the latest comprehensive taxonomic investigation of *Glochidion* from there can be dated back to Beille (1927). Recently, taxonomic studies of *Glochidion* species from Thailand (van Welzen 2007) and Vietnam (Nguyen 2007) have been conducted. In Myanmar, Kurz (1877) accepted 14 *Glochidion* species in Forest Flora of British Burma and Kress et al. (2003) recorded 33 *Glochidion* species in A Checklist of the Trees, Shrubs, Herbs and Climbers of Myanmar.

During the fieldwork in Shan State, eastern Myanmar, in December 2015, two of the authors (J. Cai and X.F. Yang) collected some Phyllantheae specimens and one belongs to *Glochidion*, which superficially differs from congeneric taxa recorded in Myanmar and its adjacent countries. After a detailed morphological investigation and herbaria examination for all the *Glochidion* species recorded in this region, it was confirmed that the species is new to science, thus it is formally described below. The new species belongs to section Glochidion, which is characterised by having three stamens in male flowers (Li 1994).

## Materials and methods

In addition to fieldwork in mountain areas of the eastern Myanmar, the present study also included analyses of *Glochidion* material from herbaria HITBC, IBSC, K, KUN, P, PE and US, as well as consideration of the taxonomic literature of China (Li 1994, Li and Gilbert 2008), India (Hooker 1887, Balakrishnan and Chakrabarty 2007, Chakrabarty and Balakrishnan 2009), Indo-China Peninsula (Beille 1927), Myanmar (Kurz 1877, Kress et al. 2003), Thailand (van Welzen 2007) and Vietnam (Nguyen 2007). Morphological analyses in the present study were performed on live plants in the field and specimens deposited in herbaria. Herbarium abbreviations follow the Index Herbarium (Thiers 2018+).

## Taxonomy

### Glochidion shanense

Taxon classificationPlantaeMalpighialesPhyllanthaceae

Gang Yao & Shixiao Luo sp. nov.

urn:lsid:ipni.org:names:77177610-1

Figures 1 , 2

#### Diagnosis.

The species is similar to *G. ellipticum* Wight in morphology, but differs from the latter by its branchlets pubescent, pedicels of female flowers 1.5–2 mm long, sepals of female flowers uniseriate and narrowly triangular, apex of style truncate slightly, persistent style of fruit stout and cylindric.

#### Type.

MYANMAR. Shan State, Pindaya, near the Htwet Ni village, West of Pindaya town, at an elevation of 1396 m, forest understory, in flowering and fruiting, 25 December 2015, *Jie Cai et al. 15CS10794* (holotype, KUN!; isotype, KUN!, IBSC!)

#### Description.

Shrubs or treelets, up to 2 m; monoecious; branchlets pubescent. Leaf blade oblong or elliptic, 9–13.5 × 4.5–6.5 cm, papery, slightly leathery, with apex acuminate to round and base broadly cuneate, sparsely pubescent along veins adaxially, pubescent abaxially; midvein and 6–9-paired lateral veins elevated abaxially. Petiole 3–4 mm long, pubescent. Stipules narrowly triangular, 2–4 mm long, pubescent. Male flowers: pedicels 6–10 mm long, densely tomentose; sepals 6, biseriate, oblong or ovate, densely tomentose; stamens 3. Female flowers: in axillary clusters, pedicels 1.5–2 mm long, densely strigose; sepals 6, uniseriate, narrowly triangular, densely strigose; ovary depressed globose, 4–5-locular, densely strigose; style connate into a cylinder, ca. 1 mm long, slightly truncate at apex, apex 4–5-lobed. Capsules depressed globose, 8–9 mm in diameter, ca. 4 mm high, sparsely pubescent, 8–10-grooved; persistent style cylindric, ca. 1 mm long; fruiting pedicels 4–5 mm long, stout, densely pubescent; seed laterally compressed, orange.

#### Distribution and habitat.

This new species is currently known only from its type locality, Shan State, eastern Myanmar, where it grows in the broadleaved and coniferous dry forest dominated by *Docynia indica* (Wall.) Decne., *Schima wallichii* (DC.) Korth. and *Pinus* species.

#### Etymology.

*Glochidion shanense* is named after its type locality, Shan State in eastern Myanmar.

#### Taxonomic discussion.

The species resembles *Glochidion ellipticum* Wight, a species widely distributed from eastern Himalaya to Taiwan Island, but differs from the latter by its branchlets densely pubescent (Figure 2G), pedicels of male flowers tomentose, pedicels of female flowers densely strigose and up to 1.5–2 mm long (Figures 2A, D), sepals of female flower uniseriate and narrowly triangular in shape (Figures 2A, D), ovaries 4–5-locular, style cylindric and truncate at apex (Figure 2D), fruits 8–9 mm in diameter and 8–10-grooved (Figure 2G), persistent styles cylindric and ca. 1 mm long (Figure 2G). In contrast, *G. ellipticum* has the branchlets glabrous (Figures 2B, H), pedicels of male flowers glabrous (Figure 2H), female flowers sessile (Figures 2B, E), sepals of female flowers biseriate and oblong in shape (Figure 2B), ovaries 3–4 (5)-locular, style columnar to columnar-conical (Figures 2B, E), fruits 6–8(rarely up to 10) mm in diameter and shallowly 6–8 (rarely 10)-grooved (Figure 2H), persistent styles obscure or slightly elevated but far less than 1 mm long (Figure 2H).

**Figure 1.**
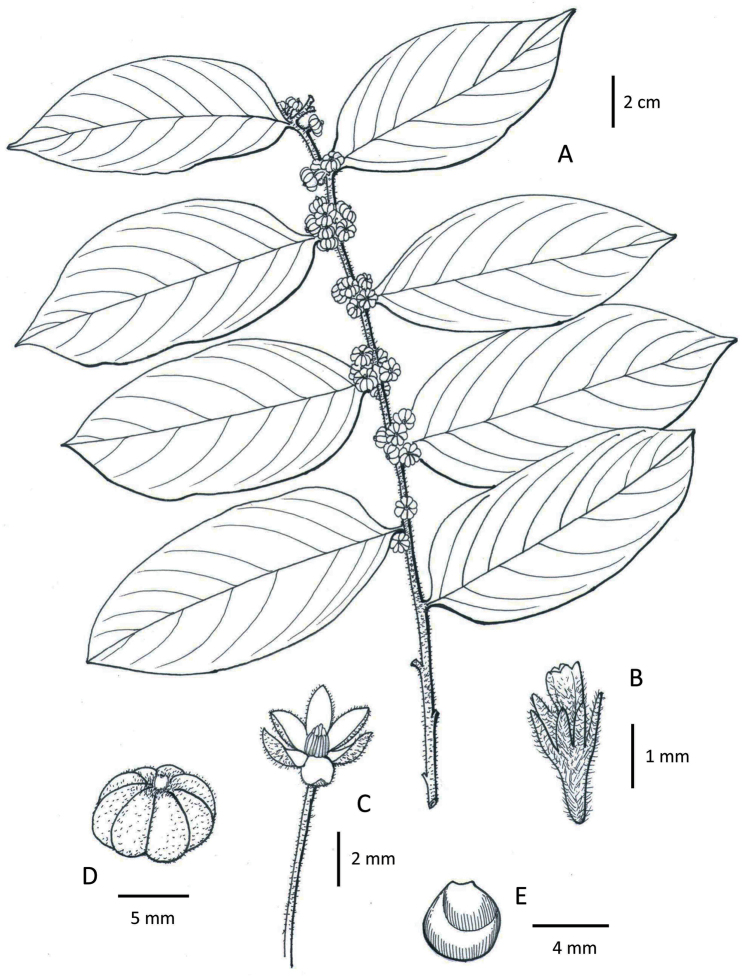
Line drawing of *Glochidion shanense* Gang Yao & Shixiao Luo, sp. nov. **A** Habit **B** Female flower **C** Male flower **D** Fruit **E** Seed. Draw by Ling Wang on *Jie Cai et al. 15CS10794* (KUN).

**Figure 2.**
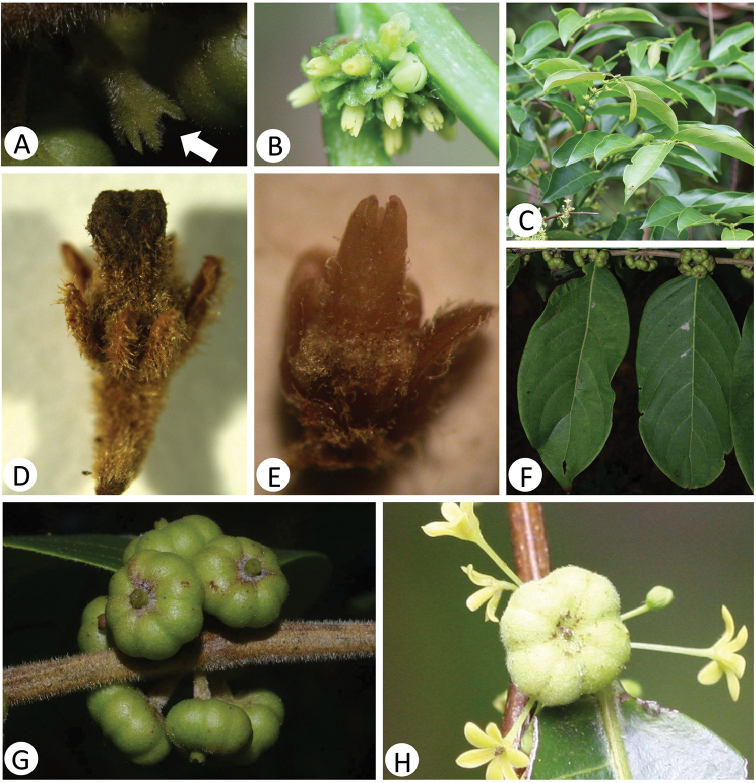
Morphological comparisons between *Glochidion ellipticum* Wight (**B, C, E, H**) and *G. shanense* Gang Yao & Shixiao Luo (**A, D, F, G**). **A, B, D, E** Female flower **C, F** Branchlets **G, H** Fruit.

## Supplementary Material

XML Treatment for Glochidion shanense
